# Decay of self-incompatibility within a lifespan in *Physalis acutifolia* (Solanaceae)

**DOI:** 10.1007/s00497-024-00517-7

**Published:** 2025-01-29

**Authors:** Chelsea Pretz, Erica Au, Stacey D. Smith

**Affiliations:** Department of Ecology and Evolutionary Biology, University of Colorado, 1900 Pleasant Street, Boulder, CO 80309 USA

**Keywords:** *Physalis*, Reproductive strategy, Self-compatibility, Self-incompatibility, Solanaceae

## Abstract

**Key message:**

Self-incompatibility decays with age in plants of *Physalis acutifolia*, and plants that have transitioned to selfing produce fewer seeds but with comparable viability. Self-compatibility in this system is closely related to flower size, which is in turn dependent on the direction of the cross, suggesting parental effects on both morphology and compatibility.

**Abstract:**

The sharpleaf groundcherry, *Physalis acutifolia*, is polymorphic for self-compatibility, with naturally occurring self-incompatible (SI) and self-compatible (SC) populations. Moreover, SI individuals have been documented to transition to SC with age, at least in greenhouse conditions. Here we tested whether this within-lifespan transition occurs predictably (developmental decay of SI) or could result from a lack of pollination (a plastic response). Using greenhouse crosses, we demonstrated that SI *P. acutifolia* plants transition to SC after 70 days, regardless of pollination treatment, consistent with predictable developmental decay. This loss of SI corresponds to a loss of pollen inhibition, with self-pollen often reaching the ovary after 24 h. The originally SI plants that transition to SC can produce viable seeds from self crosses, albeit significantly fewer than from outcrosses of SI plants or from lines fixed for SC. Throughout the experiment, we observed that flower size, which differs between SI and SC populations, was highly correlated with the compatibility phenotype. These findings suggest that the mechanisms leading to the loss of SI during a lifespan are similar to those involved in fixed losses of SI, but that older plants that transition to SC do not present the same reproductive capacity as fixed selfers.

**Supplementary Information:**

The online version contains supplementary material available at 10.1007/s00497-024-00517-7.

## Introduction

The transition from self-incompatibility (SI) to self-compatibility (SC) has been studied at varying scales, from ancient shifts involving entire families to recent changes within populations. Across all seed plants, self-compatibility, the ability to create seed from self pollen, occurs at a low rate of about 10–15% (Goodwillie et al. 2005; Wright et al. 2013). Within the tomato family Solanaceae, the evolutionary transition to self-compatibility is very common, with over half of all species being SC (Goldberg et al. 2010), resulting from over 60 losses of SI (Igic et al. 2008). Below the species level, there is a wide spectrum of variation, from fixed differences in compatibility across populations (Rick and Tanksley 1981) to mixed mating systems within populations (Broz et al. 2017; Stone et al. 2006). There is also one documented system in which individuals transition from SI (the ability to recognize, reject, or abort self pollen) to SC over their lifespan (Pretz and Smith 2022), a phenomenon that is infrequently studied but is known from other taxa (Goodwillie et al. 2004). This study investigates the transition to SC within a lifespan, the predictability and rate of the transition, and the short-term consequences of that transition for seed production and viability.

The consequences of SI to SC transitions at higher scales, such as between species or populations, have been extensively studied. In the short term, the evolution of self-compatibility may often be advantageous, for example, by facilitating range expansion (Broz et al. 2017; Levin & Miller 2021) and providing reproductive assurance (Goodwillie et al. 2005). However, evolutionary losses of SI often lead to dead ends via elevated extinction rates (Igic et al. 2008; Goldberg et al. 2010). This evolutionary outcome may be tied to inbreeding (Wright et al. 2013) and decreased genetic diversity (Stebbins 1974). In the larger picture, this combination of factors suggests that SC lineages may have high turnover, frequently arising due to short-term benefits, but also experiencing higher extinction rates as their genetic diversity erodes over time.

The transition from SI to SC during an individual’s lifespan has been studied in multiple species, although the range of consequences is not well understood. The breakdown of SI systems within a lifetime can occur as flowers age (Richardson et al. 1990; Goodwillie et al. 2004; Liao et al. 2016) and across the whole plant as it ages (Travers et al. 2004; Pretz and Smith 2022). We term this predictable breakdown ‘phenotypic decay’ to differentiate this transition from plasticity in compatibility, a more widely studied phenomenon (Pretz and Smith 2023). Previous studies have shown that the SC phenotype can be plastically induced due to heat (Ronald and Ascher 1975), salt concentration (Yang et al. 2018), and even pollen limitation (Travers et al. 2004). Importantly, plastic changes may have some potential to revert (Prabha et al. 1982) while the decay of SI with time appears irreversible, likely due to the loss of expression of genes required to block self-pollen growth (Richardson et al. 1990; Pretz and Smith 2022).

The present study focuses on the patterns and consequences of the decay of SI in *Physalis acutifolia*, a member of the tomatillo genus in the tomato family. Previous work demonstrated that this species has gametophytic self-incompatibility (Pretz and Smith 2022) in which pollen tubes start to grow down the stylar tissue, and self-pollen growth is inhibited due S-RNase proteins (Takayama and Isogai 2005). However, *P. acutifolia* is polymorphic for SI, with some populations containing SI individuals and others SC (Pretz and Smith 2022). This variation in compatibility is coupled with classic selfing sydrome traits, with the SC plants showing smaller flowers, smaller anther-stigma distance, and lower pollen:ovule ratios (Pretz and Smith 2022). We also observed that the SI individuals transition to SC with age and begin to produce fruits from self-fertilization. This transition was associated with the lack of inhibition of self-pollen tube growth and the loss of S-RNase expression (Pretz and Smith 2022), although our experiments did not exclude the involvement of other pollen and pistil factors (e.g., Goldraij et al. 2006; Ma et al. 2021). While we know that *P. acutifolia* plants have the capacity to transition from SI to SC, the time to transition, or even if all plants will eventually transition, remains in question.

The goals of this study are to better understand the predictability of this lifetime transition from SI to SC in *P. acutifolia* as well as its consequences for reproductive output*.* We first tracked the timing of the transition and examined the possible impact of pollination, given that a lack of pollination has been observed to accelerate the transition to SC in other Solanaceae (Travers et al. 2004). We also tested how patterns of pollen tube growth relate to the transition, and in particular, if SI plants that transition to SC show self-pollen tubes reaching the ovules. Lastly, we compared the fruit and seed set of ‘transitioned’ plants to those that are fixed for SC, with the prediction that transitioned plants may be poor selfers, producing fewer or low quality seed as they are older plants and the SI source populations may not have purged deleterious variation (Arunkumar et al. 2015).

## Methods & materials

### Study system and crossing design

**Fig. 1:**
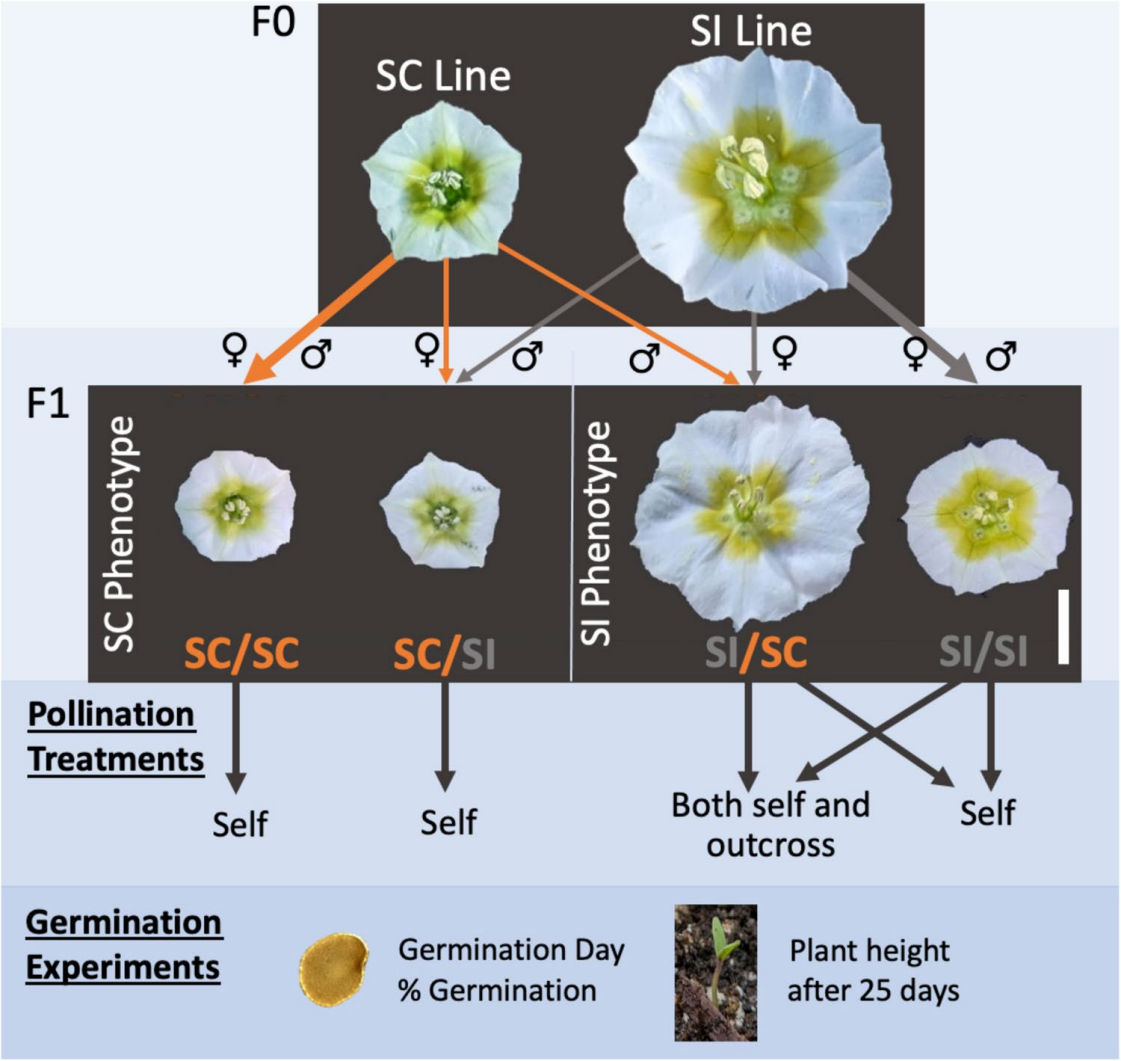
Experimental design. This experiment started with seeds from F0 crosses made in Pretz and Smith (2022) to create SI and SC lines of *P. acutifolia*. These lines were crossed to create F1s, and we tracked the direction of the cross (i.e., which parent was maternal/paternal). These individual F1s varied in flower size and compatibility phenotype, which followed maternal phenotype and were used in experimental crosses. Seeds from self-crosses and outcrosses of these were then used in a germination experiment to determine the quality of the offspring

*Physalis acutifolia* is an annual plant that displays both self-incompatible and self-compatible phenotypes (Pretz and Smith 2022). Crosses were conducted by Pretz and Smith (2022) with originally sourced seed from SI populations from New Mexico and Arizona along with a single geographically isolated SC population from Arizona discovered by C. Pretz during field surveys. The SI plants used in this experiment were offspring of crosses between SI populations, and the SC plants were from self-crosses of the SC population. In addition to these SI and SC lines, we created F1 hybrids to explore potentially intermediate compatibility phenotypes. Thus, our experiment includes four genotypes (maternal genotype/paternal genotype): both parents that were self-compatible (SC/SC), parents that differed in self-compatibility and self-incompatibility (SC/SI and SI/SC), and lastly, where both parents were self-incompatible (SI/SI) (Fig. 1). The purpose of this crossing design was to have the plants start with a range of levels of S-RNase expression, from little to no expression in SC/SC plants to high expression in SI/SI plants (Pretz and Smith 2022). A total of 88 individuals were grown in the greenhouse, comprising 28 SC/SC, 28 SI/SI, 16 SC/SI, and 16 SI/SC.

After each individual plant started to flower, we carried out hand-pollinations with self and outcross pollen. The SC/SC and SC/SI individuals, which all show the self-compatible phenotype (see results), were self-pollinated to produce selfed offspring for comparison in seed germination experiments (Fig. 1). The SI/SC and SI/SI individuals were all self-incompatible, and we split them among pollination treatments. For half of the SI/SC individuals and half of the SI/SI individuals, we carried out both self-crosses and outcrosses to test the effect of cross-pollination on the transition to SC; this will be referred to as the ‘both’ treatment. The other half were only selfed (Fig. 1). For the outcrosses, we used a mixture of pollen from flowering individuals to minimize any patterns specific to a single pollen source.

### Predictability in transition rate to SC

For the initially self-incompatible individuals (SI/SC and SI/SI, Fig. 1), we conducted crosses every two weeks to determine when they transitioned. If there was successful fruit set from a self-cross, this was considered the transition date. We compared the timing of the transition between the both and self pollination treatments to test if this transition is delayed due to outcrossing. The times to transition were standardized relative to the first day of flowering.

### Pollen tube growth before and after the transition to SC

We measured pollen tube growth at multiple points during the transition to SC in SI phenotype individuals to test the prediction that self-pollen would travel farther toward the ovary post-transition. We collected self-pollinated styles after 24 h, fixed them in 70% EtOH, stained them in aniline blue, and mounted them on a slide following Pretz and Smith (2022). In vivo stained pollen tubes were then imaged on Olympus IX81 Inverted Widefield Microscope with the Olympus cellSens Dimension V3.2 64-bit program and processed using ImageJ/Fiji (Schindelin et al. 2012). The distance the pollen tubes traveled was measured as a percentage of the length of the style. Since the individuals transitioned at different rates, the date of the cross was taken as a percentage from the first day of flowering and the transition date. For example, styles collected from a cross taken halfway between the first day of flowering and the date of transition to SC would be considered the 50% timepoint. We measured pollen tube growth for four individuals of SI/SI genotype and four SI/SC genotypes at four time points.

### Quantity and quality of SC offspring from transitioned SI plants

To compare the quantity and quality of offpsring across treatments and genotypes, we collected fruits and extracted seeds. In addition to the hand-pollinations, we tagged flowers that received no treatment and collected fruits from self-fertilization (‘tagged’). Fruits from the tagged treatments have similar numbers of seeds as those that were manually self-pollinated (see results) and thus were included in the comparisons. Seeds from each fruit were counted, and to avoid pseudoreplication, an average seed set was taken for each individual for the selfed (including tagged) treatments and the outcrossed treatments (when available) (Fig. 1).

We measured seed germination in order to assess seed viability across genotypes, treatments and, for originally SI plants, before and after the transition to SC. For this experiment, we focused on the SI/SI and the SC/SC plants. Four to eight randomly drawn seeds from 20 successful crosses were grown from the SC/SC and and SI/SI genotypes and for both pre- and post-transition crosses for SI/SI, for a total of 456 seeds. For the pre- and post-transition germination experiment, we used the same 10 individuals to provide a direct comparison. Germination date (from only plants that germinated) and plant height (roots to shoot tip) after 25 days after germinating were measured. We calculated the expected number of viable seeds for each treatment by multiplying the average seed set by the germination rate.

### Correlation and predictive traits

During the experiment, we noticed a difference in flower size between genotypes and measured that difference to assess its possible relationship to selfing. We measured three flowers from each individual from images using ImageJ/Fiji. We used the average for each individual in downstream analyses.

### Statistical analyses

We conducted pairwise comparisons of the time to transition across treatments and genotypes. We first used a Shapiro–Wilk test to assess normality. Transition times were normally distributed for both and self pollination treatments (pooling across SC/SI and SI/SI genotypes), so an unpaired *t*-test was conducted. However, transition times were non-normal within genotypes, so a Mann–Whitney *U* Test was conducted.

The seed set and germination data also deviated from normality and thus we used a Kruskal–Wallis test to compare means across the treatments and genotypes, followed by a Wilcoxon Comparison with Bonferroni correction to determine the directional differences. We also calculated Spearman’s correlations between flower size and seed traits and applied the Bonferroni correction to adjust the significance for multiple tests.

A mixed model was used to test the relationship between the transition to SC and the amount of self-pollen tube growth, treating individual as a random effect. We compared linear, expositional, quadratic, and square root models and chose the model with the lowest AIC (see Online Resource 1 for set of models compared).

All statistical analyses were performed in the R environment (R Development Core Team 2012). The following R-packages were used: lme4 (Bates et al. 2015), dplyr (Wickham et al. 2022, plyr (Wickham 2011), ggplot2 (Wickham 2016), sjPlot (Lüdecke 2022), tidyverse (Wickham et al. 2019).

## Results

### Predictability in transition rate to SC

**Fig. 2:**
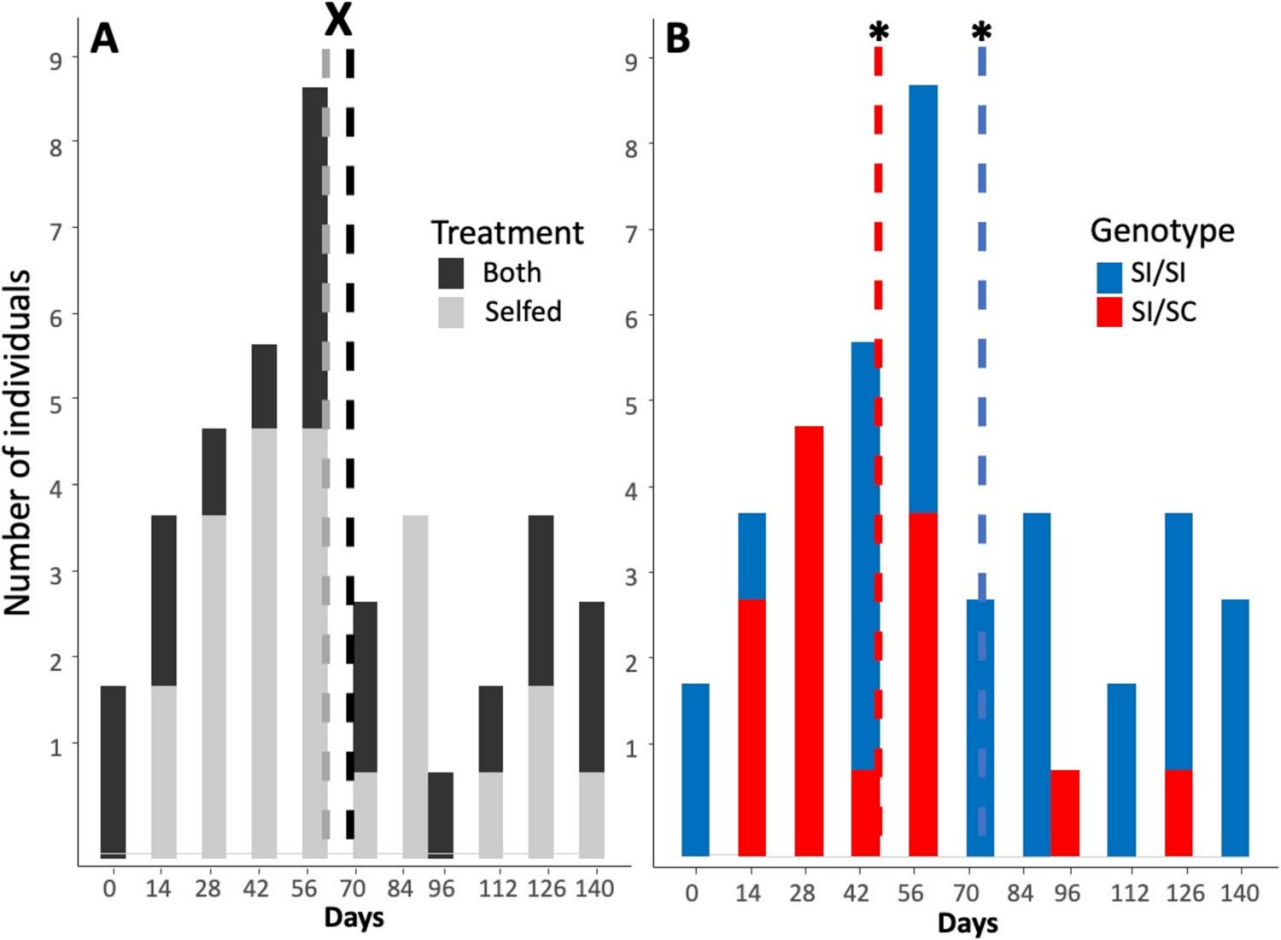
Days until originally self-incompatible plants from the SI/SI and SI/SC genotypes transitioned to SC. The same data are depicted in both panels, with the bars broken by treatment (both self and outcrosses vs. self only) in (**A**) and by genotype in (**B**). The vertical dashed shows the mean times to transition for each group. The ‘Both’ treatment is represented by 18 individuals and the SelfOnly by 25 days. In (**B**), the SI/SI and SI/SC genotypes are represented by 28 and 15 individuals, respectively. The “X” represents no statistical difference, while “*” denote lines that are statistically different with the threshold of *p* < 0.05

Our results show that early outcrossing does not influence the speed of transition from SI to SC, but the genotype does (Fig. 2). We only considered individuals with over a 5% successful interspecific cross rate, reducing the original number from 22 to 18 for the both treatment (full dataset in Supporting Information–Online Resource 2). We found no statistical difference (F = 0.53, df = 24, *P* = 0.15) in the average number of days to transition between plants that were both self and outcrossed (69 ± 11 days; n = 18 individuals) and those that were only selfed (61 ± 7 days; n = 25). However, we did see a signficant difference between the genotypes (U = 122, df = 35, *P* = 0.025). On average SI/SC plants took 47 days (± 8) to transition, with 15 individuals measured, while SI/SI individuals took 74 days (± 8) to transition, with 28 measured.

### Pollen tube growth before and after the transition to SC

**Fig. 3:**
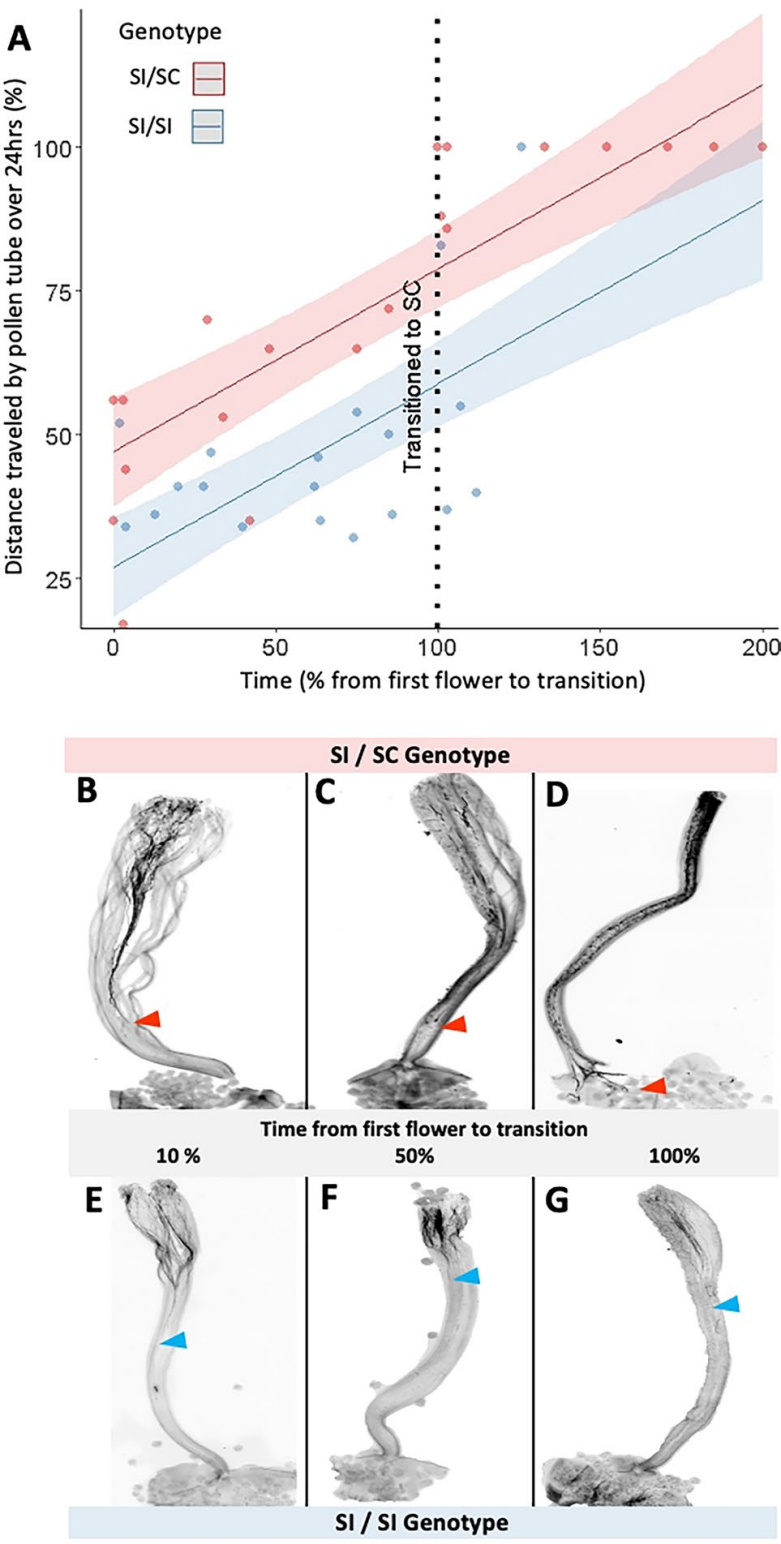
Time series of pollen tube growth in vivo. **A** The distance the pollen tubes traveled after 24 h relative to the total length of the style is shown as a percentage on the y-axis. Time on the x-axis is measured as a percentage of the transition towards SC, with 0% being the first day of flowering and 100% being the first successful self-pollination. Pollen tube growth was also examined beyond 100% (after the transition) for comparison. **B**–**G** Pollen tube growth at different time points ~ 10%, 50%, and 100% time to transitioning from the first day of flowering. **B**–**D** Pollen tube growth in an SI/SC individual with the red arrow pointing to the end of the pollen tube. **E**–**G** Pollen tube growth in an SI/SI individual with the blue arrow pointing to the end of the pollen tube

We observed a predictable increase in the distance traveled by self-pollen as the originally SI plants transitioned to self-compatibility (see full dataset in Online Resource 3). At the beginning of an individual’s lifespan, the pollen tube growth for an originally SI plant terminates within the top ca. 60% of the style tissue (Fig. 3A). As the plant ages, the inhibition of the pollen tubes weakens until the pollen tubes can continue through the style tissue. Our linear model indicated a significant difference in pollen tube growth between the SI/SC and SI/SI genotypes (Appendix S5). Specifically, self pollen tubes are arrested earlier in SI/SI plants than in SI/SC plants (Fig. 3B-G). Moreover, while most self-pollen tubes reach the ovary after the transition to SC in the SI/SC phenotype, we observed three out of four instances were SI/SI plants achieved self-pollination despite traveling less than 50% of the distance to the ovary in the first 24 h after pollination (Online Resource 3). This suggests that, while self pollen eventually reaches the ovary, it takes longer than 24 h to achieve fertilization.

### Quantity and quality of SC offspring from transitioned SI plants

**Fig. 4:**
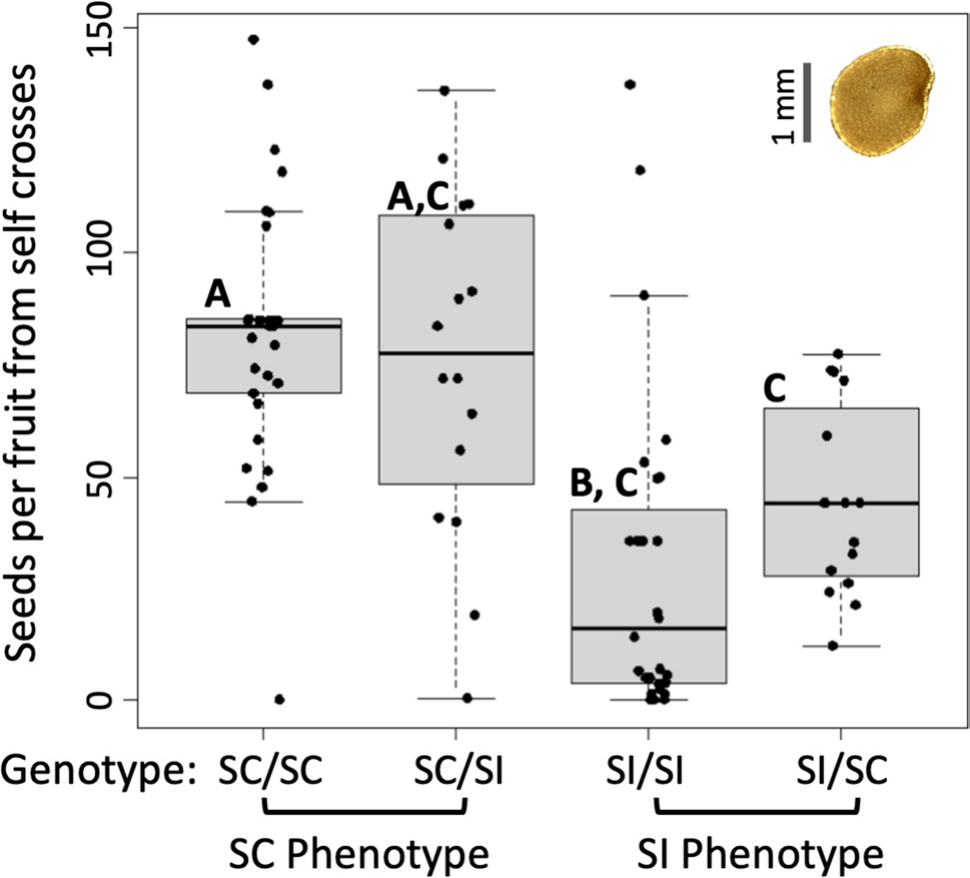
Selfed seed set from self-fertilization after all individuals have transitioned. Medians are shown for each of the four genotypes with a wide horizontal line. The boxes denote the upper and lower quartiles. The top right corner is an image of an average seed with a scale bar of 1 mm

Once a plant with the SI phenotype transitions to self-compatibility, it produces seeds, but fewer than those produced by plants that began as self-compatible (see full dataset in Online Resource 4). After the transition from SI to SC, the SI/SI plants produced only 30 (± 7) seeds per fruit on average from self-crosses, which is much lower than that from outcrossing (85 ± 6). Similarly, the SI/SC plants (also originally self-incompatible) produced only 44 (± 5) seeds per fruit on average from self-crosses (Fig. 4). As expected, the self-compatible lines showed higher fruit set from self-crosses, with 76 (± 9) seeds per fruit on average for SC/SI plants and 82 (± 5) for SC/SC plants (Table 1). We found a significant effect of genotype on treatment (Kruskal–Wallis χ2 = 32.69, df = 3, *P* = 3.731e-07). Looking pairwise across the genotypes, the SI/SI seed set is significantly different from SC/SC (*P* = 1.6e − 05) and SC/SI (*P* = 0.00301), and SI/SC is significantly different from SC/SC (*P* = 0.00021) (Fig. 4).

**Table 1:** Seed set and germination from self- and outcrosses of SI/SI plants compared to SC/SC plants

Treatment/Genotype	Average seed weight (mg)	Average seed set per Fruit	Number of seeds tested (Number of individuals)	Average days to germination	Average percent germination (%)	Expected average viable seeds
Outcrossed/SI/SI	1.21 (± 0.9)	85 (± 6)	152 (N = 11)	11.21 (± 0.35)	65	56 (± 4)
Selfed/SI/SI	1.19 (± 0.5)	30 (+ 7)	143 (N = 20)	11.5 (± 0.46)	73	26 (+ 5)
Selfed/SC/SC	1.08 (± 1.3)	82 (± 5)	160 (N = 20)	7.84 (± 0.24)	84	69 (± 5)

Overall expected average quantity of viable seeds informed by germination experiment. The average seed set is the same as Fig. 3; expected viable seeds are the average seed set multiplied by the germination rate. Seeds were germinated from several different individuals to estimate the germination rate

Our germination experiment revealed that seed quality differed between SI and SC plants but not between SI plants pre- and post-transition. While seeds from all the crosses were similar in weight, the seeds from SI plants (both outcrosses pre-transition and self-crosses post-transition) were slower to germinate and had lower percentage of germination success compared to seeds from SC plants (Table 1; Online Resource 5). These differences across the genotypes (SI/SI and SC/SC) for number of days to germinate and percent germination were highly significant (Kruskal–Wallis, *P* ≤ 0.0005 for both).

Despite these differences in seed weight and germination rate, there were no statistically significant differences in plant height across genotypes and treatments after 25 days post-germination (Kruskal–Wallis χ2 = 3.6017, df = 2, *P* = 0.1652). The average height of seedlings grown from self crosses of SC/SC plants was 23.05 (± 0.44) cm versus 24.30 (± 0.49) cm for outcrosses of SI/SI and 23.70 (± 0.75) cm for self crosses of SI/SI (post-transition).

When considering the seed set along with the germination rate, we find that transitioned SI plants produce fewer viable seeds overall than SI plants pre-transition and than SC plants. Even though the germination rate for selfed seeds of the SI/SI plants was only slightly lower than for selfed SC/SC plants (and higher than for outcrossed SI/SI plants), the number of seeds per fruit is less than half of that for the other treatments, resulting in the lowest number of expected viable seeds (Table 1).

### Correlated and predictive traits

**Fig. 5:**
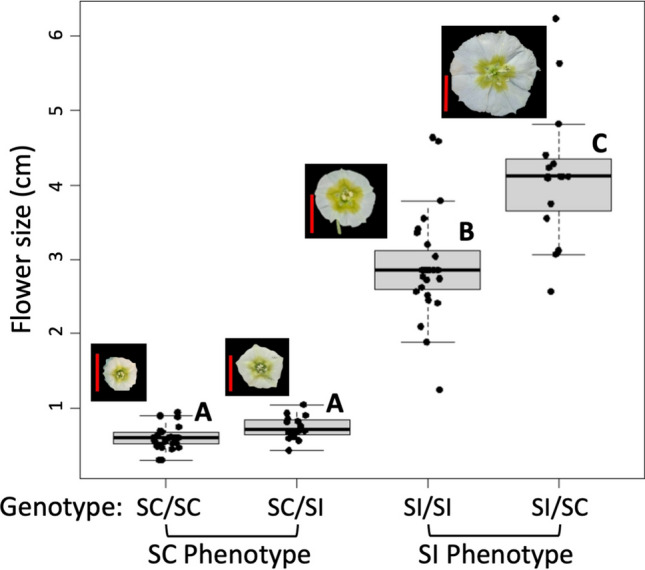
Floral size by genotype. The boxes depict the upper and lower quartiles around the median (wide horizontal bar). I. Example flowers are shown from each genotype. The inset red scale bar represents 1 cm

In our experiment, we found that flower size in the F1 plants followed their maternal phenotype and that this, in turn, affected the breeding system phenotype. The SC/SC and SC/SI plants both had small flowers like their self-compatible maternal line, while the SI/SC and SI/SI plants had large flowers (Fig. 1). The average flower size for each genotype was 0.60 (± 0.18) cm for SC/SC, 0.74 (± 0.04) cm for SC/SI, 4.13 (± 0.23) cm for, SI/SC, and 2.90 (± 0.13) for SI/SI: (Fig. 5). All pairs except for SC/SC and SC/SI were statistically different (‘SC/SC’/’SI/SI’, *P* = 5.2e−10; ‘SC/SC’/’SI/SC’, *P* = 2.3e−07; ‘SI/SI’/’SC/SI’, *P* = 2.6e−07; ‘SI/SI’/’SI/SC’, *P* = 0.00027; and ‘SC/SI’/’SI/SC’, *P* = 9.1e−06). Consistent with our findings above, the SC/SI plants tended to behave like SC/SC plants and the SI/SC plants like SI/SI plants (Fig. 5). Overall, we found strong correlations between flower size, fruit size, seed set and days to transition across the entire dataset (Table 2).

**Table 2:** The correlations between flower size and reproductive traits across the genotypes

Trait	Average flower size	Average fruit size	Average seed set	Days to transition
Average flower size (cm)	–	2.33E−04	1.27E−05	3.30E−15
Average fruit size (Tagged) (cm)	− 0.44	–	4.59E−08	1.36E−10
Average seed set (Tagged) (#)	− 0.49	0.58	–	1.26E−06
Days to transition	0.74	− 0.64	− 0.53	–

The bottom diagonal shows the values for Kendall’s τ (a non-parametric dependence test) and the upper diagonal shows the Bonferroni-corrected *p*-values between the pairs. We included only values for the ‘tagged’ untreated flowers, whose fruit and seed set comes from self-fertilization

## Discussion

### The predictable transition to SC is not influenced by pollination

The transition to SC was caused by the decay of SI over time and not by plasticity due to pollination. While outcrossing delays the onset of self-fertilization in *Solanum carolinense* (Travers et al. 2004), in our study, we saw that outcrossed pollen did not change the rate of transition to SC. The SI plants that were both self- and outcrossed had similar means to time to transition as those that only received self-pollen (Fig. 2). Nevertheless, we did observe some variation in the speed of the transition across genotypes. For example, although all individuals eventually transitioned, the SI/SC individuals transitioned nearly a month faster than the SI/SI genotype (47 vs. 74 days, on average). These results hint that there is a threshold for S-RNase necessary for a functional self-incompatible phenotype (Qin et al. 2006), and, in our experiment, that the F1 genotype, which presumably has less S-RNase expression, transitions faster. Our previous work (Pretz and Smith 2022) implicates S-RNase expression as a major determinant of the SI phenotype, and differences in the behavior of S-RNases of different *S*-alleles (Qi et al. 2023) could also contribute to the wide variation in the speed of transition (Fig. 2), if some only weakly inhibit the growth of self-pollen tubes.

In line with these observations, we found that the transition from SI to SC in an originally SI plant is directly related to the rate of self-pollen growth in the style. We saw that self pollen tube growth varied between SI/SC and SI/SI individuals, having different intercepts but the same slopes (Fig. 3). At time zero (first day of flowering, when the plants are self-incompatible), self pollen tubes grow nearly 50% of the distance from the stigma to the ovary in SI/SC plants after 24 h, compared with only 25% in SI/SI plants. This indicates a lower barrier for self-pollen in SI/SC styles at the start of their lifespan compared to SI/SI plants as the distance a pollen tube can travel is associated with the strength of the inhibition (Covey et al. 2010). After the transition to self-compatibility, we observed that self pollen was able to completely reach the ovary after 24 h in the SI/SC plants while in the SI/SI plants, the tubes had still not reached the ovaries (Fig. 3). Given that the self-pollination was successful in both cases, we conclude that the self-pollen must have eventually reached the ovary in the SI/SI plants, albeit at a much slower rate than in the SI/SC plants. While this pattern of faster self-pollen growth in SI/SC plants implicates differences in the strength of SI in the pistil (e.g., lower inhibitory levels of S-RNase in the SI/SC genotype), our experimental design does not exclude changes in the pollen that might also occur with senescence.

Our study uncovered additional differences across the F1 lines that suggest complex inheritance with some strong maternal component. In particular, having a self-compatible maternal parent (with the small-flowering selfing phenotype, Pretz and Smith 2022) and self-incompatible paternal plant results in offspring with small flowers (Fig. 1), which quickly transition to SC (starting from the first day of flowering), and produce large numbers of seeds from self-crosses (Fig. 4). Crosses in the opposite direction show the opposite trends (SI/SC offspring show large-flowers and seed set like their SI maternal parent, Fig. 1). We also observe an unexpected pattern whereby the largest flowers among the F1s belong to the SI/SC plants as opposed to the SI/SI plants (Figs. 1, 5). There are known cases of parental effects on flower size and morphology (Leino et al. 2003), but we are unaware of examples involving the modification of compatibility. In addition to influences through the maternally inherited organellar genomes, genomic imprinting is another possible mechanism that could explain the observed parental effects (Hatorangan et al. 2016). The joint maternal effects on flower size and compatibility might also suggest pleiotropy (shared genetic architecture of the two traits), but studies in other systems suggest that the two are not genetically correlated (McElderry et al. 2022). Thus, dissecting these complex parental effects on flower size and compatibility in *P. acutifolia* will require additional in-depth genetic studies, considering both organellar and nuclear genotypes.

### Understanding the short-term consequences of transitioning to SC

In addition to examining the rates of transition with respect to genotype and pollination treatment, this study also sought to examine the reproductive output of transitioned plants. We found that SI plants produced higher numbers of seed pre-transition (from outcrossing) than they produced post-transition (from selfing), although the quality of the seeds was similar in terms of weight and germination rate (Table 1). We also observed wide variation in seed set from self-crosses of the SI/SI plants, suggesting that even ‘transitioned’ plants that can self are not all effective at selfing. Thus, self-incompatible plants can transition to SC for reproductive assurance and the quality of these offspring is similar, but their number of potential offspring from self-fertilization is reduced. In addition to these short-term consequences of selfing, we expect that long-term reliance on selfing would have additional evolutionary implications due to inbreeding (Stebbins 1974; Wright et al. 2013).

### Broader evolutionary impacts caused by the transition to SC

While this experiment was conducted in a greenhouse with an ideal growing environment, some consequences could translate into nature. The typical growing season of *P. acutifolia* is two months long, ranging from July to August (Sullivan 2004), and we would have to double this growing season to four months for the plants to transition to SC. Still, such long-lived plants are likely to occur in human-dominated habitats where water is available for much of the year, e.g., due to irrigation (C. Pretz, pers. obs.) and the ability to transition to self-compatibility would allow reproduction even as pollinator abundance varies. This flexibility might also help expand the range (Broz et al. 2017; Grossenbacher et al. 2017; Koski et al. 2019). If this is to occur, these individuals will likely encounter potentially new abiotic and biotic stressors. Interestingly, the loss of SI in Solanaceae has been associated with changes in defense strategies, with SC taxa evolving towards more inducible and herbivore-specific responses (Campbell and Kessler 2012).

## Conclusion

This study contributes to our understanding of how an individual transitions to self-compatibility within a lifespan. In *Physalis acutifolia,* the SI system predictably decays over an individual’s lifetime rather than due to environmental stimuli. Transitioned *P. acutifolia* plants will produce viable seeds, although fewer seeds are produced per fruit. However, the speed of this transition to SC depends on the genotype, with individuals with two SI parents transitioning more slowly than hybrids. The precise *S*-alleles might also contribute to variation in the rate of transition from SI to SC, although this possibility remains to be explored. This study highlights the benefits of a short-term transition to SC in that SI plants can produce viable seeds, albeit fewer of them. While species that rely on selfing might be destined for extinction in the long run, the flexibility and fluidity to transition to SC on a smaller scale might be particularly advantageous with the immediate benefit of reproductive assurance. The transition from SI to SC has been studied in many systems and at different scales, but there is much we could learn by narrowing our focus to better understand lifetime developmental transitions such as those studied here.

## Supplementary Information

Below is the link to the electronic supplementary material.

Supplementary file1 (XLSX 5 kb)

Supplementary file2 (XLSX 58 kb)

Supplementary file3 (XLSX 13 kb)

Supplementary file4 (XLSX 19 kb)

Supplementary file5 (XLSX 33 kb)
